# Effect of lateral wedge-shaped orthopedic insole on patients with genu varus: A protocol for systematic review and meta-analysis

**DOI:** 10.1371/journal.pone.0274789

**Published:** 2022-09-16

**Authors:** Zhongyi Deng, Xinwei Yang, Xiaochuan Li, Xiali Xue, Huiqiang Luo, Gaitian Wu, Luyuan Zeng, Yun Qi, Ning Li

**Affiliations:** 1 Institute of Sports Medicine and Health, Chengdu Sport University, Chengdu, Sichuan, PR China; 2 The Third Affiliated Hospital of SUN YAT-SEN University, Guangzhou, Guangdong, PR China; 3 Nanchong Gaoping Traditional Chinese Medicine Hospital, Nanchong, Sichuan, PR China; 4 School of Sports Medicine and Health, Chengdu Sport University, Chengdu, Sichuan, PR China; 5 The Third People Hospital of Chengdu, Chengdu, Sichuan, PR China; Mugla Sitki Kocman Universitesi, TURKEY

## Abstract

**Background:**

Genu varus (GV) is a common deformity characterized by leg bending, which seriously threatens human health. At present, there is no definite conclusion to explain the cause of genu varus. The purpose of this study is to systematically review and meta-analysis the effectiveness and scientific basis of the clinical efficacy of customized orthopedic insoles on genu varus and hope to provide a reference for future research in this field.

**Methods:**

The following electronic databases will be searched from inception to January 2022: Pubmed, Cochrane Library, MEDLINE, EMBASE, Web of Science, Weipu, Wanfang Data, and CNKI. Randomized controlled trials (RCTs) were collected to examine the effect of lateral wedge-shaped orthopedic insole on patients with genu varus. We will consider inclusion, select high-quality articles for data extraction and analysis, and summarize the intervention effect of lateral wedge orthotic insoles on patients with genu varus. Two reviewers will screen titles, abstracts, and full texts independently according to inclusion criteria; Data extraction and risk of bias assessment were performed in the included studies. We will use a hierarchy of recommended assessment, development, and assessment methods to assess the overall certainty of the evidence and report findings accordingly. Endnote X8 will be applied in selecting the study, Review Manager 5.3 will be applied in analyzing and synthesizing.

**Results:**

The results will provide evidence for judging the effect of lateral wedge-shaped orthopedic insole on patients with genu varus.

**Conclusion:**

Our study will provide reliable evidence for the effect of lateral wedge-shaped orthopedic insole on patients with genu varus.

**Trail registration:**

**INPLASY registration number:** INPLASY202190002 https://www.google.com/search?client=firefox-b-d&q=INPLASY202190002.

## Introduction

Genu varus (GV) is a common deformity characterized by leg bending, which seriously threatens human health. The incidence of genu varus is about 10% [1]. At present, there is no definite conclusion to explain the cause of GV. However, there are relevant literature reports that in addition to congenital genetic malformations [2], Acquired factors such as vitamin D deficiency [3], weight [4,5], sport events [6] are relevant to GV. Some studies have shown that GV affects people’s posture and movement mode, causes the force imbalance between the inner and outer space of the knee joint, which may eventually lead to knee osteoarthritis (KOA) [7] or Anterior cruciate ligament injury [8]; GV not only affects the knee itself but also has a complex impact on the biomechanics of the lower limbs. Jeong Bo et al. found that patients with GV are often accompanied by foot compensatory changes such as subtalar joint rotation and arch collapse, which leads to the change of biological force line of lower limbs and finally systemic symptoms [9]. GV can also affect motor performance, Isın, A [10], used a digital caliper. He used a digital caliper to measure the quadriceps angle values and the intercondylar ankle distance of male football players and ordinary peers, determined that the prevalence of GV and abnormal quadriceps angle in adolescent football players was significantly higher than that in their non-athlete peers. Aiyegbusi, et al. [11] found that GV was significantly correlated with the prevalence and severity of Achilles tendinopathy in 85 Nigerian High-level Track and field athletes. At present, reliable treatment strategies for GV include surgical correction, rehabilitation exercise, and External orthosis for lower limbs, but GV still seriously affects people’s quality of life.Therefore, it is necessary to pay more attention to genu varus.

The orthopedic insole is a special insole customized after analyzing the biomechanical parameters such as plantar pressure and gait of patients’ lower limbs, The design principle of orthotic insole includes adjusting the stress center of the sole [12], balancing plantar pressure [13], supporting arch [14], improving foot cushioning [15]. At present, it has been widely used in the prevention, treatment, and rehabilitation of various foot and lower limb diseases. Chapman, et al. [16] found that the lateral wedge orthotic insole reduces the adduction torque of the external knee, thus, it is helpful for patients with KOA, which often occurs in the group of patients with GV. Studies have also shown that supporting transverse wedge insoles can minimize the increase of ankle or subtalar valgus torque, therefore, it has a certain regulatory effect on the correction of GV [17]. Some scholars [18] have followed up the patients with varus deformity of KOA for two years and found that orthopedic insoles may restrict the progression of degenerative articular cartilage lesions of KOA.After the manufacture of orthopaedic insoles, patients only need to be instructed to wear them as required, and simple maintenance of insoles at ordinary times can achieve the effect of intervention. It eliminates the trouble of wearing external fixed orthosis and the inferiority complex in mind. For some people who can not carry out rehabilitation exercise to correct and postpone genu varus, orthopaedic insoles are the best choice before surgical treatment of knee varus.

Other literature reports state the contrary evidence that wearing a transverse wedge insole for 12 months does not provide any symptomatic or structural benefit compared with a flat control insole [19]. Lateral wedge insoles worn for 12 months provided no symptomatic or structural benefits compared with flat control insoles. The guidelines for the treatment of KOA formulated by the American College of Rheumatology and the osteoarthritis research society do not use orthopedic insoles as the recommended treatment [20].

Therefore, the role of customized orthopedic insoles in patients with GV needs decisive evidence. The purpose of this study is to systematically review and meta-analysis the effectiveness and scientific basis of the clinical efficacy of customized orthopedic insoles on GV and hope to provide a reference for future research in this field.

## 2. Methods

### 2.1. Study registration

The study aims to evaluate the effectiveness of the effect of lateral wedge-shaped orthopedic insole on patients with GV. We will use the preferred reporting items for systematic reviews and meta-analysis statements to guide our systematic evaluation report. And the review was registered in the international platform of a registered systematic review and meta-analysis protocols (INPLASY) database (INPLASY202190002).

### 2.2. Eligibility criteria

#### 2.2.1 Types of participants

For all patients with Symptomatic or asymptomatic genu varus, no restrictions will be applied in terms of age, sex, race, country, and disease.

#### 2.2.2 Types of interventions

The experimental group wore the outer wedge orthopedic insoles.

#### 2.2.3 Types of comparator

The comparator received a placebo or other treatment technique.

#### 2.2.4 Types of outcome measures

The Primary Prognostic Indicatorsare VAS pain Scale. And the secondary prognostic indicators are symptoms or symptom scores Plantar pressure analysis, Gait analysis.

#### 2.2.5 Types of study

We will include randomized controlled trials of the effect of lateral wedge-shaped orthopedic insole on patients with genu varus.

### 2.3. Data sources

Comprehensive retrieval databases include the following databases: Pubmed, Cochrane Library, EMBASE, Web of Science, Weipu, Wanfang Data, and China National Knowledge Infrastructure (CNKI), The retrieval time is from the establishment of the database to January 2022.

### 2.4. Retrieval strategy

The search terms on PubMed are as follows: Orthopedic insole (e.g., “Foot Orthoses” or “Orthoses, Foot” or “Foot Orthosis” or “Orthosis, Foot”); Genu Varum (e.g., “Bow Leg” or “Bow Legs” or “Genu Varum” or “Knee Varus”); randomized controlled trial (e.g., “randomized” or “clinical trial”). Combinations of Medical Subject Headings (MeSH) and text words will be used. The search strategy will be developed in pubmed and translated to the other databases. These search terms are shown in Table 1.

**Table 1:** Search strategy for the PubMed database.

Number	Search items
#1	Orthopedic insole
#2	Foot Orthoses
#3	Orthoses, Foot
#4	Foot Orthosis
#5	Orthosis, Foot
#6	Foot Orthotic Devices
#7	Device, Foot Orthotic
#8	Devices, Foot Orthotic
#9	Foot Orthotic Device
#10	Orthotic Device, Foot
#11	Orthotic Devices, Foot
#12	Foot Arch Supports
#13	Arch Support, Foot
#14	Arch Supports, Foot
#15	Foot Arch Support
#16	Support, Foot Arch
#17	Supports, Foot Arch
#18	Orthotic Shoe Inserts
#19	Insert, Orthotic Shoe
#20	Inserts, Orthotic Shoe
#21	Orthotic Shoe Insert
#22	Shoe Insert, Orthotic
#23	Shoe Inserts, Orthotic
#24	Orthotic Insoles
#25	Insole, Orthotic
#26	Insoles, Orthotic
#27	Orthotic Insole
#28	#1 or #2- #27
#29	Genu Varum
#30	Bow Leg
#31	Bow Legs
#32	Leg, Bow
#33	Legs, Bow
#34	Genu Varus
#35	Knee Varus
#36	**#**29 or #30- #35
#37	Randomized controlled trial
#38	Randomized
#39	Clinical trial
#40	#37 or #38- #39
#41	#28 and #36 and #40

### 2.5. Data collection and analysis

#### 2.5.1. Selection of studies

**Fig 1:**
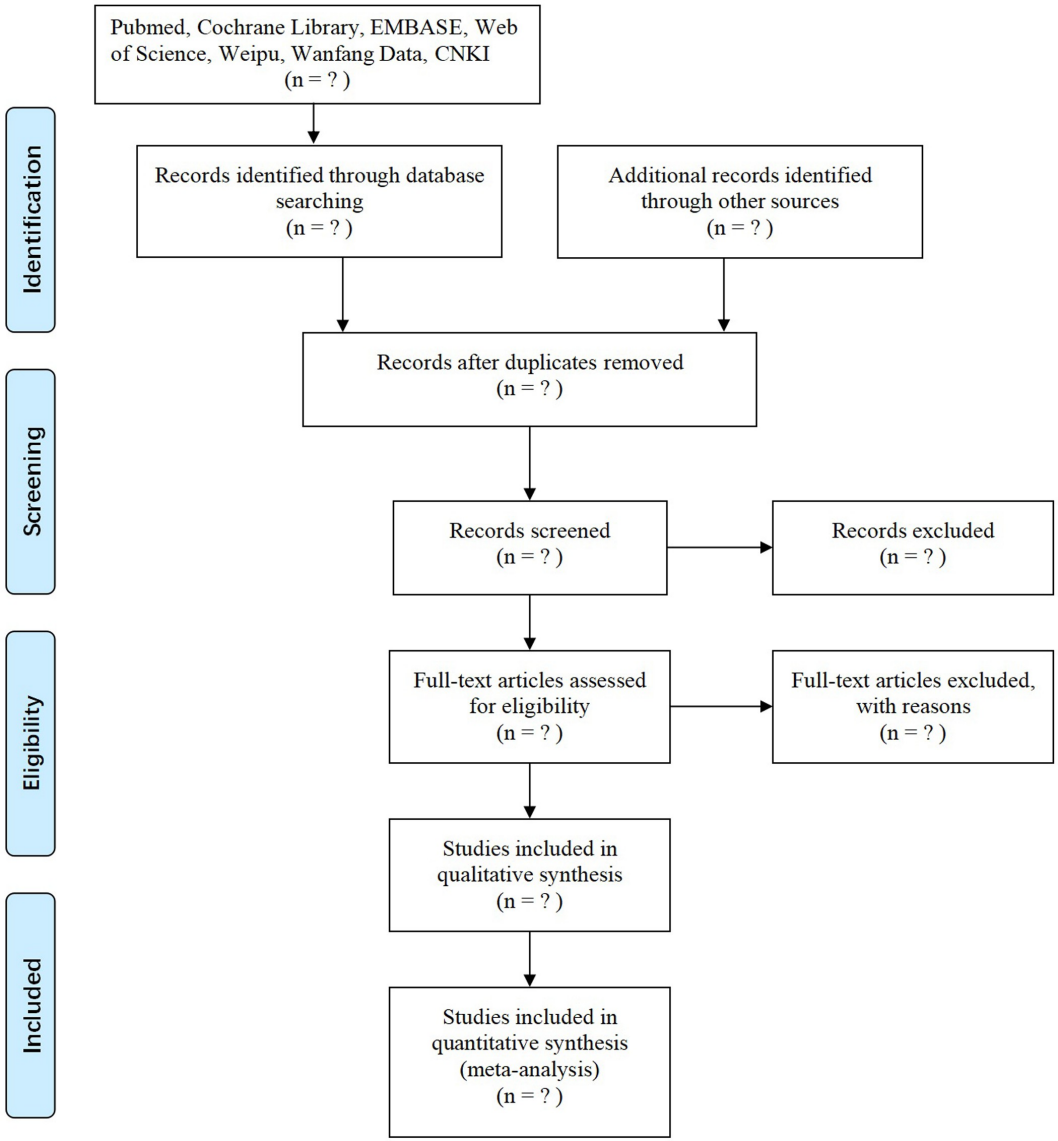
Flow diagram of the study selection process.

All investigators will be properly trained prior to the commencement of the data screening. Rayyan online literature management software (https://rayyan.qcri.org) will also be introduced to screen and manage literature. After screening a random sample of 50 citations, Kappa test will be used to calculate the inter-observer agreement for all studies included. If Kappa value is less than 0.75, a second round of training will be implemented. Duplicate publications of original research will be excluded. The titles and abstracts of identifiable articles will be screened independently by two reviewers (XWY and XCL), to exclude reports that clearly do not meet the inclusion criteria. The same reviewers will then independently examine full-text articles to determine eligibility. When disagreements arise, the third author (ZYD) will be asked to evaluate the full text and the discrepancy will be resolved by group discussions. Excluded trials and the rationale for exclusion will be recorded. Fig 1 depicts the study selection processes in a Preferred Reporting Items for Systematic Reviews and Meta-Analyses (PRISMA) flow diagram.

#### 2.5.2. Data extraction and management

The other two researchers (HQL and GTW) will extract data independently to fill out the predesigned form. The information includes author, country, publication year, methodological quality, characteristics of participants, the details of intervention and comparisons, outcomes, the specific data, results, conclusions, follow-up, adverse events, conflicts of interest, sources of funds, and ethical approval. The extracted data will be cross-checked by the two researchers. A third researcher (NL) will be involved in a disagreement occurs. The authors of the studies included will be contacted for further information when necessary.

#### 2.5.3. Assessment of risk of bias in included studies

Two authors (ZYD and LYZ) independently evaluated the risk of bias of the included studies and cross-checked the results. Disagreements were resolved by consulting a third reviewer (XCL). The quality of the included studies was assessed using the Cochrane Collaboration risk assessment tool for RCTs [21]. The risk of bias (low, unclear, or high) will be assessed based on random sequence generation, allocation concealment, blinding of participants and personnel, blinding of outcome assessment, incomplete outcome data, selective reporting, and other biases.

#### 2.5.4. Data synthesis

RevMan5.3 software will be used to integrate and analyze included studies. Dichotomous data will be reported as risk ratio (RR) with 95% confidence interval (CI), whereas continuous data will be reported as mean difference (MD) or standard mean difference (SMD) with 95% CI. Results of the meta-analysis will be visualized by forest plots. We will combine more than one trial to estimate pooled intervention effect using the meta-analysis when studies examine the same intervention and outcomes with comparable methods in similar populations. We will pool the continuous data using the inverse variance method and dichotomous data using the Mantel-Haenszel method. We will use the fixed-effect model to combine data when statistical heterogeneity is low. However, when P<0.1 or I^2^>50%, the random-effect model will be used to provide a more conservative estimate of effect. If a meta-analysis is not possible, we will provide a narrative summary of the results from individual studies.

#### 2.5.5. Measures of treatment effect

In this protocol, we will use a 95% CI to rigorously analyze the dichotomous data. And for the continuous data, weight means difference or standard mean difference is used to measure the efficacy of 95% CI. Skewed data and non-quantitative data will be presented descriptively.

#### 2.5.6. Dealing with missing data

We will attempt to contact the authors to request the missing or incomplete data. If those relevant data are not acquired, they will be excluded from the analysis.studies without data will still be included in the narrative synthesis.

#### 2.5.7. Assessment of heterogeneity

Heterogeneity is influenced by many factors (e.g., age, different intervention forms, missing data, etc), and we plan to evaluate its degree through the χ^2^ test and *I*^*2*^test. Meanwhile, We decided to select the model for Meta-analysis according to the significance of statistical heterogeneity,and tau statistics and 95% prediction intervals are also reported. If *I*^*2*^ statistic <50% and P-value > 0.05, we will use the fixed-effect model to combine data. However, when P<0.05 or I^2^≥50%, the random-effect model will be used to provide a more conservative estimate of effect. For the heterogeneity of Q-value statistics, we mainly look at the p value. P value > 0.1 had no heterogeneity, P value <0.1 had heterogeneity. Furthermore, we will conduct subgroup analysis or sensitivity analysis to investigate potential sources of heterogeneity.

#### 2.5.8. Subgroup analysis

If sufficient comparable studies are available, subgroup analysis will be conducted in terms of age, sex, intervention forms, treatment course.

### 2.6. Sensitivity analysis

Sensitivity analysis will be performed according to sample size, study design, heterogeneous quality, methodological quality and statistical model, the trials with quality defects will be excluded to ensure the stability of the analysis results.

### 2.7. Publication bias analyses

We will detect publication biases and poor methodological quality of small studies using funnel plots if 10 or more studies are included in the meta-analysis.

### 2.8. Grading the quality of evidence

This paper will use the evidence quality rating method to evaluate the results obtained from this analysis. GRADE will be assessed across the domains of risk of bias, consistency, directness, precision, and publication bias. In the context of the system review, quality reflects our confidence in the effectiveness of the assessment. It has 4 evaluation levels, namely, high (further research is very unlikely to change our confidence in the estimate of effect), moderate (further research is likely to have an important impact on our confidence in the estimate of effect and may change the estimate), low (further research is very likely to have an important impact on our confidence in the estimate of effect and is likely to change the estimate), or very low (very uncertain about the estimate of effect) [22]. GRADE will be used on each individual outcome.

### 2.9. Ethical and dissemination

It requires no ethical approval for this study based on collecting and collating documents. Findings will be disseminated through a peer-reviewed publication.

## 3. Discussion

GV is a common disease at any age, it not only seriously affects the work and life of patients, but also causes depression and anxiety. At present, the main methods for the treatment of GV are surgery [23] and rehabilitation exercise [24], As an effective external auxiliary technology, lateral wedge orthotic insole has become a new choice for the prevention and treatment of GV. However, due to the large age span of GV, different evaluation and diagnosis methods for GV patients, and the complexity of lower limb biomechanics. In the actual treatment process, the most suitable insole type and insole making method for patients with GV cannot be selected, so the treatment effect of the insole has not been widely recognized. Therefore, the results of this review will provide real and reliable effectiveness for the effect of lateral wedge orthotic insole on the GV group.

The study also has some defects as follows: low quality of original researches, The difference length of wearing insoles, various duration of disease different dosage, and intervention cycle, language restriction, wear of insole during wearing, etc. All of these will lead to some bias and influence the results of evaluation results, ultimately affecting this study’s reliability.

## Supporting information

S1 Checklist PRISMA-P (Preferred Reporting Items for Systematic Review and Meta-Analysis Protocols) 2015 checklist: Recommended items to address in a systematic review protocol. (DOC)

S1 File (DOCX)

## Abbreviations

KOA: Knee osteoarthritis
GV: Genu varus
RCTs: Randomized controlled trials
