# Supplementary material for: Effect of lateral wedge-shaped orthopedic insole on patients with genu varus: A protocol for systematic review and meta-analysis
Source: PLoS One. 2022 Sep 16;17(9):e0274789. doi: 10.1371/journal.pone.0274789 (PMC9481019; doi:10.1371/journal.pone.0274789)
Supplement: S1 File — (DOCX) [file pone.0274789.s002.docx]

Search strategy in PubMed

| **Number** | **Search items** |
| --- | --- |
| #1 | Orthopedic insole |
| #2 | Foot Orthoses |
| #3 | Orthoses, Foot |
| #4 | Foot Orthosis |
| #5 | Orthosis, Foot |
| #6 | Foot Orthotic Devices |
| #7 | Device, Foot Orthotic |
| #8 | Devices, Foot Orthotic |
| #9 | Foot Orthotic Device |
| #10 | Orthotic Device, Foot |
| #11 | Orthotic Devices, Foot |
| #12 | Foot Arch Supports |
| #13 | Arch Support, Foot |
| #14 | Arch Supports, Foot |
| #15 | Foot Arch Support |
| #16 | Support, Foot Arch |
| #17 | Supports, Foot Arch |
| #18 | Orthotic Shoe Inserts |
| #19 | Insert, Orthotic Shoe |
| #20 | Inserts, Orthotic Shoe |
| #21 | Orthotic Shoe Insert |
| #22 | Shoe Insert, Orthotic |
| #23 | Shoe Inserts, Orthotic |
| #24 | Orthotic Insoles |
| #25 | Insole, Orthotic |
| #26 | Insoles, Orthotic |
| #27 | Orthotic Insole |
| #28 | #1 or #2- #27 |
| #29 | Genu Varum |
| #30 | Bow Leg |
| #31 | Bow Legs |
| #32 | Leg, Bow |
| #33 | Legs, Bow |
| #34 | Genu Varus |
| #35 | Knee Varus |
| #36 | **#**29 or #30- #35 |
| #37 | Randomized controlled trial |
| #38 | Randomized |
| #39 | Clinical trial |
| #40 | #37 or #38- #39 |
| #41 | #28 and #36 and #40 |

Search strategy in MEDLINE

1. #1 exp Genu varus/
2. #2 (knee varus $ or varus knee).tw.
3. #3 1 OR 2
4. #4 (lateral wedge-shaped orthopedic insole or orthopedic insole).tw
5. #5 #3 and #4

Search strategy in Embase

1. #1 Genu varus:ti,ab OR knee varus :ti,ab OR or varus knee:ti,ab
2. #2 lateral wedge-shaped orthopedic insole:ti,ab OR orthopedic insole:ti,ab
3. #3 #2 AND #3

Search strategy in Cochrane

#1 Genu varus OR knee varus* OR avarus knee

#2 (lateral wedge-shaped orthopedic insole OR orthopedic insole*)

#3 #1 AND #2
